# Lidocaine coinfusion alleviates vascular pain induced by hypertonic saline infusion: a randomized, placebo-controlled trial

**DOI:** 10.1186/s12871-021-01329-2

**Published:** 2021-04-10

**Authors:** Zhiping Song, Shibiao Chen, Yang Zhang, Xiaoyun Shi, Na Zhao, Zhengyu Liao

**Affiliations:** 1 Department of Anesthesia, The First Affiliated Hospital of Nanchang University, No.17 Yongwaizheng Street, Nanchang, Jiangxi 330006 People’s Republic of China; 2 Department of Orthodontics, The Affiliated Stomatological Hospital of Nanchang University, No.49 Fuzhou Road, Nanchang, Jiangxi 330008 People’s Republic of China; 3 The Key Laboratory of Oral Biomedicine, Jiangxi Province, No.49 Fuzhou Road, Nanchang, Jiangxi 330008 People’s Republic of China

**Keywords:** Lidocaine, Coinfusion, Vascular pain, Hypertonic saline

## Abstract

**Background:**

Hypertonic saline solution has been frequently utilized in clinical practice. However, due to the nonphysiological osmolality, hypertonic saline infusion usually induces local vascular pain. We conducted this study to evaluate the effect of lidocaine coinfusion for alleviating vascular pain induced by hypertonic saline.

**Methods:**

One hundred and six patients undergoing hypertonic saline volume preloading prior to spinal anesthesia were randomly allocated to two groups of 53 each. Group L received a 1 mg/kg lidocaine bolus followed by infusion of 2 mg/kg/h through the same IV line during hypertonic saline infusion; Group C received a bolus and infusion of normal saline of equivalent volume. Visual analogue scale (VAS) scores of vascular pain were recorded every 4 min.

**Results:**

The vascular pain severity in Group L was significantly lower than that in Group C for each time slot (*P* < 0.05). The overall incidence of vascular pain during hypertonic saline infusion in Group L was 48.0%, which was significantly lower than the incidence (79.6%) in Group C (*P* < 0.05).

**Conclusion:**

Lidocaine coinfusion could effectively alleviate vascular pain induced by hypertonic saline infusion.

**Trial registration:**

Chinese Clinical Trial Registry, number: ChiCTR1900023753. Registered on 10 June 2019.

## Background

Hypertonic saline solution has been frequently utilized for fluid resuscitation, management of hyponatremia, and reducing intracranial pressure, among other procedures. In anesthetic practice, hypertonic saline is one alternative for volume preloading before spinal anesthesia. In some institutions in China, hypertonic saline with a concentration not exceeding 3% is allowed to be infused via peripheral veins. However, due to the nonphysiological osmolality, hypertonic saline infusion usually induces local vascular pain. Patients may experience the sensation of stinging and compression around the site of venous cannulation, which reduces patient satisfaction.

Lidocaine is a widely used local anesthetic agent. It can reduce propofol injection pain when administered in the same vein, and it can also decrease pain when hypertonic saline is injected for sclerotherapy, implying its local anesthetic effect on local vein [1, 2]. Lidocaine has both central analgesic and anti-inflammatory effects. Recently, intravenous lidocaine infusion has drawn great attention as an analgesic adjunct. Continuous IV lidocaine infusion resulting in a stable blood concentration could decrease intraoperative opioid requirements and relieve postoperative pain [3, 4]. We hypothesized that simultaneous infusion of lidocaine into the same venous line during hypertonic saline infusion might be effective for alleviating the vascular pain since lidocaine might act via both local anesthetic and central analgesic effects. This study was designed to evaluate the efficacy and safety of lidocaine coinfusion for relieving vascular pain induced by hypertonic saline infusion.

## Methods

This clinical trial was approved by the China Ethics Committee of Registering Clinical Trials (ChiECRCT20190219) and was registered in the Chinese Clinical Trial Registry, www.chictr.org.cn (Number: ChiCTR1900023753) prior to patient enrollment. The study was conducted in accordance with CONSORT guidelines. No changes were made to the protocols or analyses after the trial commenced. After obtaining written informed consent from all patients, we conducted this prospective, double-blinded, randomized, controlled trial in the First Affiliated Hospital of Nanchang University from October 2019 to March 2021. Inclusion criteria: aged 18–65, American Society of Anesthesiologist (ASA) classes I and II, scheduled for elective surgery requiring spinal anesthesia. Patients with chronic pain, abnormal sensation, neurologic deficits, and allergy to lidocaine were excluded.

An attending anesthesiologist from the First Affiliated Hospital of Nanchang University, who was independent of patient management and data collection, generated random numbers (in a 1:1 ratio) using SAS 9.2 software (SAS Institute, Cary, NC, USA). The results of randomized allocation were sealed in sequentially numbered opaque envelopes. A total of 106 patients were randomly allocated into two equal groups: a lidocaine group (Group L) and a placebo control group (Group C). All the subjects received 200 ml of 3% hypertonic saline via peripheral vein for volume preloading before spinal anesthesia. Patients in Group L received a bolus of 1 mg/kg lidocaine at the initiation of hypertonic saline infusion, followed by continuous infusion of lidocaine at the speed of 2 mg/kg/h. In Group C, normal saline was used as placebo; patients in Group C received a bolus of normal saline at the initiation of hypertonic saline infusion, followed by continuous infusion of normal saline, and the volume of normal saline was equal to the volume of lidocaine in Group L.

Patients were fasted for 6 h before anesthesia and given no preoperative sedatives. Monitoring of ECG, noninvasive blood pressure (BP) and pulse oximetry (SpO_2_) was performed for all patients on their arrival at the anesthesia preparation room. A nurse who was in charge of drug preparation, opened an opaque envelope, delivered a preprepared syringe (filled with 50 ml of 1% lidocaine solution or normal saline) into the room, and then left. Therefore, the medical staff and patients in the anesthesia preparation room were blinded to the allocation. A 22-gauge i.v. cannula was inserted into the vein on the dorsum of the hand. To maintain hemodynamic stability, 200 ml of 3% hypertonic saline was administered for volume preloading prior to spinal anesthesia. Hypertonic saline was infused at the speed of 10 ml/min controlled by an electronic infusion pump. Meanwhile, in Group L, lidocaine was simultaneously infused into the same venous line through a 3-way stopcock and an infusion extension line. The dosage of lidocaine was a bolus of 1 mg/kg (0.1 ml/kg) followed by continuous infusion of 2 mg/kg/h (0.2 ml/kg/h), which was administered by a microinfusion syringe pump. Lidocaine coinfusion was stopped when the 200 ml of hypertonic saline preloading was accomplished. In Group C, an equivalent volume of normal saline was coinfused. Spinal anesthesia was performed after volume preloading, followed by surgical operation.

The incidence of vascular pain was the primary outcome of this study. During the hypertonic saline infusion, vascular pain was assessed and recorded every 4 min (T_4m_, T_8m_, T_12m_, T_16m_, T_20m_) by using a visual analogue scale (VAS) score, which varied from 0 (no pain) to 10 (the worst imaginable pain). Major adverse effects, such as tinnitus, dizziness, and extravasation, phlebitis, and venous thrombosis on the cannulation site were also observed and recorded in this study. No changes were made regarding trial outcomes after the trial commenced.

In a preliminary observation, approximately 80% of patients experienced pain during hypertonic saline infusion. The sample size required to detect a 30% reduction at a level of significance of 5% and a power of 90% was 48 patients per group. Considering a dropout rate of 10%, 53 patients were included in each group. No interim analyses were made. Statistical analyses were performed using SPSS for Windows software program version 24 (SPSS, Chicago, IL, USA). Data were expressed as the mean ± SD, or number of patients (% frequency), or median (range), as appropriate. Numerical data were compared using t-test if normally distributed, or using Mann–Whitney U test if not normally distributed. Categorical data were compared using a Chi-square test or Fisher’s exact test. *P* values of less than 0.05 were considered to be statistically significant.

## Results

**Fig. 1:**
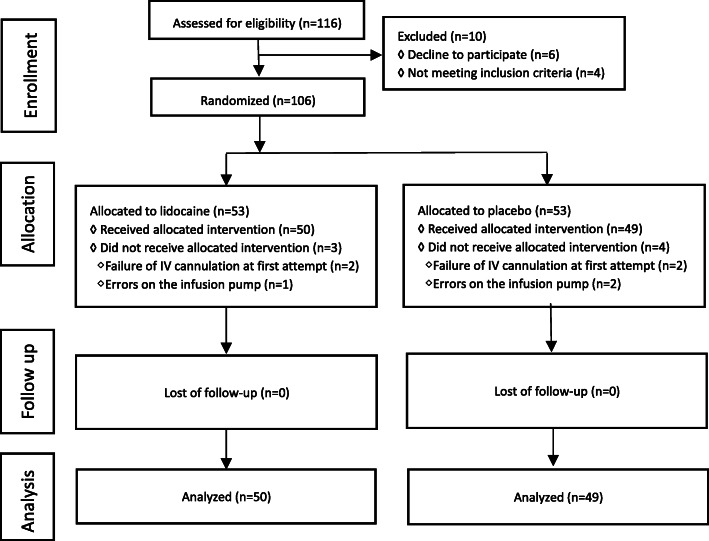
CONSORT diagram showing the flow of study participants

Subject enrollment and analysis are illustrated in Fig. 1. A total of 116 patients were assessed for eligibility for this study, and 106 subjects were enrolled. Seven patients were withdrawn due to failure of venous cannulation at the first attempt on the hand (4 patients) and operation errors on the infusion pump (3 patients). Ultimately, 99 patients (Group L = 50, Group C = 49) were analyzed in this trial. There were no significant differences (*P* > 0.05) observed between the two groups with respect to age, weight, height, gender, and ASA classification (Table 1).

**Table 1:** Demographic and clinical characteristics of two groups of patients

Item	Group L (*n* = 50)	Group C (*n* = 49)	*P* value
Age (year)	42.3 ± 9.9	42.9 ± 10.9	0.753
Weight (Kg)	63.9 ± 8.5	61.4 ± 6.7	0.113
Height (cm)	166.2 ± 5.5	164.8 ± 6.8	0.239
Gender, Female/Male	19 / 31	22 / 27	0.486
ASA class, I/II	43 / 7	45 / 4	0.356

*ASA* American Society of Anesthesiologists

Values are expressed as mean ± SD or as number of patients

**Fig. 2:**
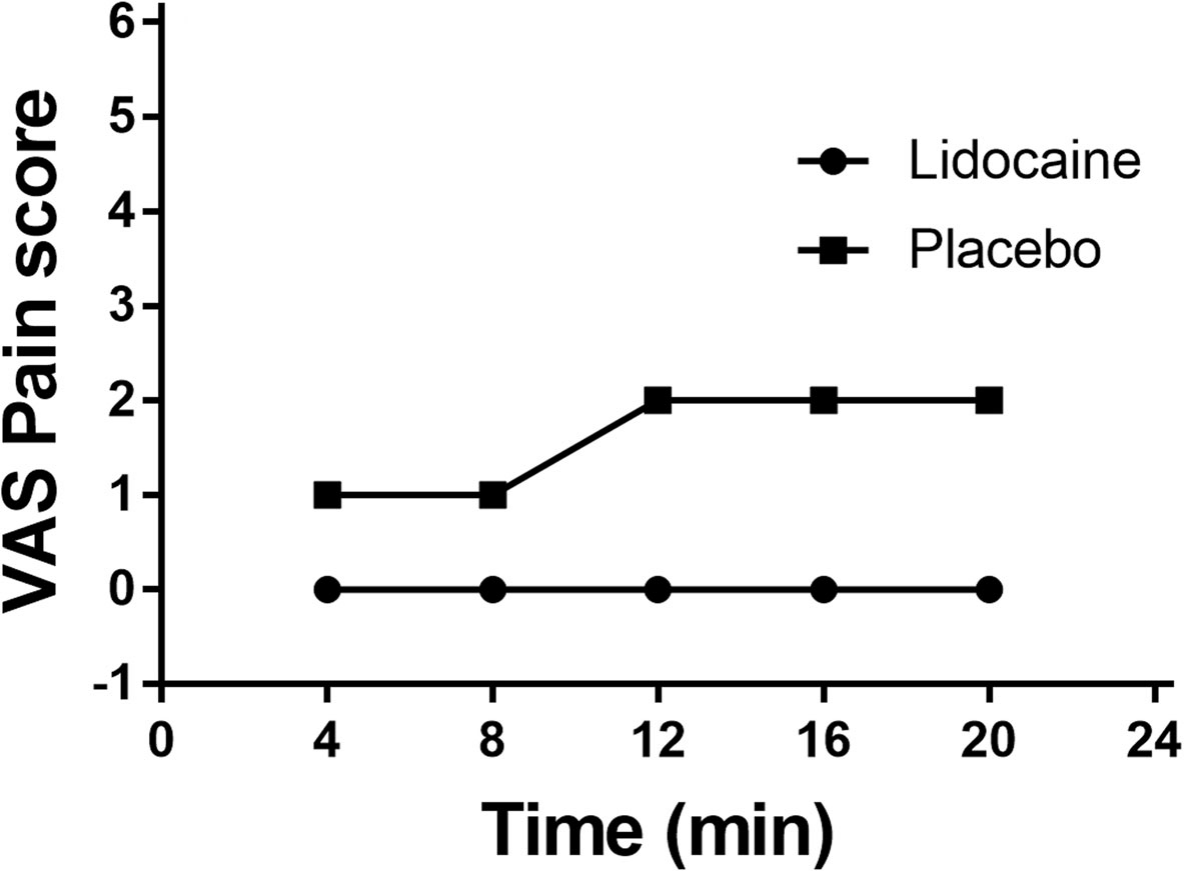
The median VAS pain scores of the two groups during hypertonic saline infusion

**Fig. 3:**
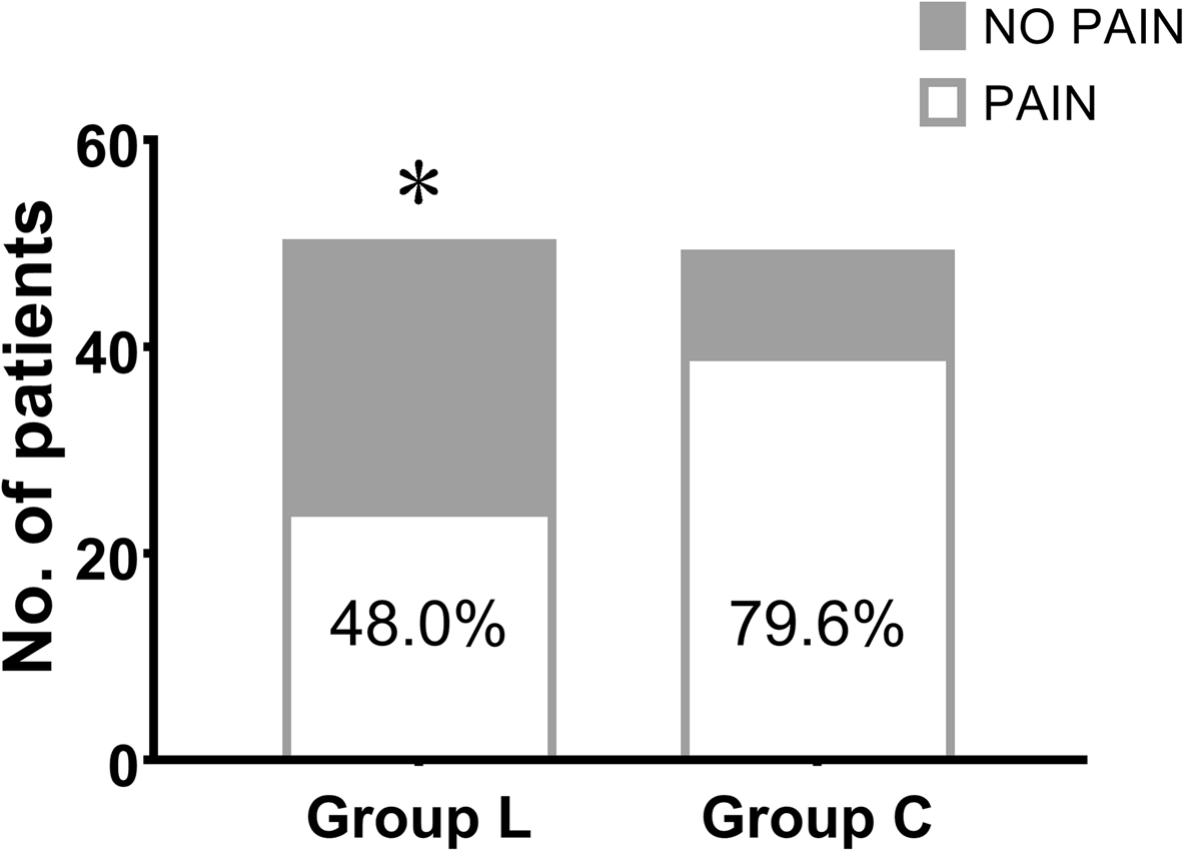
The incidence of vascular pain during hypertonic saline infusion of the two groups.^*^P = 0.001

The values of VAS pain score during the hypertonic saline infusion at each time slot were presented in Table 2 and Fig. 2. In Group L, the median VAS pain scores were 0 in each time slot, while in Group C, the median scores were both 1 in T_4m,_ T_8m_, and were all 2 in the other three time slots. The pain severity of patients in Group L was significantly lower than that in Group C in each time slot (*P* < 0.05). There were 26 (52.0%) patients out of 50 in Group L who did not experience any pain during the 20-min infusion, while there were only 10 (20.4%) patients in Group C who did not complain of any pain. The overall incidence of vascular pain in Group L was 48.0%, which is significantly lower than the incidence (79.6%) of vascular pain in Group C (Fig. 3).

**Table 2:** VAS pain score on each time slot of the two groups

Time	Group L (*n* = 50)	Group C (*n* = 49)	*P* value
T_4m_	0 (0,2)	1 (0,2)	0.002
T_8m_	0 (0,2)	1 (0,3)	0.005
T_12m_	0 (0,3)	2 (0,4)	0.005
T_16m_	0 (0,3)	2 (0,4)	0.000
T_20m_	0 (0,3)	2 (0,4)	0.000

Values of VAS pain score are presented as median (range)

The adverse events were also examined and noted in this trial. Four subjects (8%) out of 50 in Group L experienced transient tinnitus or/and dizziness during the infusion. No other complications were observed in this study.

## Discussion

Hypertonic sodium chloride solution is one of the fluids commonly used in clinical practice. It is frequently utilized for management of hyponatremia, reducing intracranial pressure, and alleviating cerebral edema associated with neurological injuries [5, 6]. Furthermore, hypertonic saline is effectual in the resuscitation of patients with hypovolemic shock [7, 8]. It is also an alternative for volume preloading before anesthesia [9], and for perioperative fluid management [10, 11].

In some institutions, it is common practice to allow only central venous line infusion of hypertonic saline due to the safety concerns associated with peripheral venous infusion such as extravasation, tissue ischemia, phlebitis, and venous thrombosis. Nevertheless, growing evidence has demonstrated that the incidence of local tissue damage was very low and the degree of injury was mild, when 3% sodium chloride solution was infused peripherally for a prolonged duration [5, 12, 13]. One study that evaluated children receiving 3% hypertonic saline via peripheral veins reported no infusion-related complications [14]. Considering the potential complications associated with central venous catheters (CVC), such as infection, hematoma, and pneumothorax, many clinicians have begun to question the necessity of central venous cannulation specifically for continuous infusion of medications with high osmolality. In our study, we adopted peripheral venous access instead of CVC for hypertonic saline infusion, and none of the 99 patients experienced any infusion-associated tissue injuries. Though it was not the primary research objective, our study did further prove the safety of peripheral intravenous infusion of 3% hypertonic saline.

Due to the nonphysiological osmolality, infusion of hypertonic solution via a peripheral vein usually induces local vascular pain. The mechanisms of vascular pain evoked by hypertonic saline have not yet been fully elucidated. It is believed that the osmolality-associated pain is mediated by nociceptors in the vessel wall [15, 16]. In the present study, 39 (79.6%) subjects out of 49 in the placebo group complained of vascular pain during the infusion. Additionally, the pain intensity was closely related to time course. The vascular pain was mild in the first few minutes and became more pronounced as the infusion went on, reaching its maximum at approximately 12 min, and then remained stable. The characteristics of vascular pain observed in our research were consistent with previous findings [15, 17].

Vascular pain usually reduces patients’ satisfaction, and alleviating vascular pain is an important issue [18, 19]. Lidocaine is a widely used local anesthetic drug that mediates its pharmacological roles mainly by blocking the sodium channels on the cell membrane. Recent studies have reported that lidocaine also has central analgesic effects by inhibiting the glycinergic system, suppressing the N-methyl-D-aspartate (NMDA) receptors, decreasing the excitability of spinal dorsal horn, and activating opioid receptors [20–22]. Previous studies have demonstrated that a bolus of low-dose (≤40 mg) lidocaine into the same vein effectively mitigated vascular pain induced by propofol injection mainly through a local anesthetic effect [1, 23]. However, the analgesic duration for vascular pain was short, just several minutes [17, 24]. Recently, intravenous lidocaine infusion has drawn great attention as an analgesic adjunct. Continuous infusion of a larger dose of lidocaine could achieve a stable plasma concentration that exerts a central analgesic effect. For example, perioperative lidocaine infusion relieved postoperative pain in several types of open abdominal and laparoscopic procedures [3, 4], and continuous infusion of lidocaine provided significant analgesia for different sorts of acute pain in the emergency department [25–28].

In the present study, lidocaine was simultaneously and continuously infused into the same vein into which hypertonic saline was infused. Therefore, lidocaine could work through the combination of both a local anesthetic effect and a central analgesic effect. The results of our study suggested that lidocaine coinfusion was effectual in relieving vascular pain associated with persistent hypertonic saline infusion. The overall incidence of pain during the 20-min infusion was 48.0% in Group L, a reduction of 32% compared with placebo group; and the median pain scores with lidocaine coinfusion were significantly lower than those in Group C in each time slot. The adverse events associated with lidocaine were mild; only 8% of the patients in lidocaine group complained of transient tinnitus or dizziness and did not require any intervention, which indicated the safety of lidocaine coinfusion. Since lidocaine is a worldwide commonly used drug and inexpensive ($0.5/100 mg in China), lidocaine coinfusion deserves further studies in reducing vascular pain associated with hyperosmolar fluid infusion.

There were some limitations in this study. First, we did not monitor the blood levels of lidocaine. The dose of lidocaine used in the current trial was based on previous studies [29, 30]. This dose has been repeatedly demonstrated to be safe, and the plasma concentration was far below the toxic level (5 μg/ml). Second, our study was a single-center trial with limited samples. Therefore, further multicenter studies with large samples are warranted.

## Conclusion

In conclusion, this study showed that 3% hypertonic saline infusion through the peripheral vein did not cause obvious local tissue injuries but induced vascular pain. Lidocaine coinfusion could effectively mitigate vascular pain during hypertonic saline infusion.

## Data Availability

The datasets used and/or analyzed during the present study are available from the corresponding author on reasonable request.

## Abbreviations

IV: Intravenous
VAS: Visual analogue scale
ASA: American Society of Anesthesiologist
SpO_2_: Pulse oximetry
BP: Blood pressure
ECG: Electrocardiogram
CVC: Central venous catheter
